# *Group B Streptococci* recto-vaginal colonization, antimicrobial susceptibility pattern, and associated factors among pregnant women at selected health facilities of Wolaita Sodo Town, Southern Ethiopia

**DOI:** 10.3389/fmicb.2023.1277928

**Published:** 2023-10-30

**Authors:** Abera Kumalo, Biruk Gebre, Shimelis Shiferaw, Wokil Wolde, Tamirayehu Shonde

**Affiliations:** ^1^Department of Medical Laboratory Science, College of Health Sciences and Medicine, Wolaita Sodo University, Wolaita Sodo, Ethiopia; ^2^Department of Medical Laboratory Science, Wolaita Sodo University Comprehensive Specialized Hospital, Wolaita Sodo, Ethiopia; ^3^Department of Obstetrics & Gynecology, School of Medicine, College of Health Sciences and Medicine, Wolaita Sodo University, Wolaita Sodo, Ethiopia; ^4^School of Medicine, College of Health Sciences and Medicine, Wolaita Sodo University, Wolaita Sodo, Ethiopia

**Keywords:** *GBS*, antibiotic susceptibility pattern, pregnant women, Wolaita Sodo, Ethiopian

## Abstract

**Background:**

*Streptococcus agalactiae* or *Group B Streptococcal* colonization of the gastrointestinal and genital tracts of pregnant women usually remains asymptomatic, even though it is the critical determinant of infection in neonates and young infants. It causes early and late onset of invasive *Group B Streptococcus (GBS)* disease manifesting as septicemia, meningitis, and pneumonia. Now it is recognized as an important cause of maternal and neonatal morbidity and mortality in many parts of the world including Ethiopia, where the magnitude of the problem has been little studied. The aim of this study was to assess the prevalence of *GBS* colonization and to identify associated risk factors and antimicrobial susceptibility patterns among pregnant women at selected health facilities of Wolaita Sodo Town, Southern Ethiopia.

**Methodology:**

A health-facility-based cross-sectional study design was conducted at WSUCSH & Wolaita Sodo Health Center from June to August, 2022. A total of 279 pregnant women who were in ANC follow-up at 35–37 weeks of gestation were included. For *GBS* isolation, recto-vaginal swabs were inoculated in 1 mL Todd-Hewitt broth medium supplemented with 10 μg/mL colistin and 15 μg/mL nalidixic acid, followed by identification of isolates based on colonial morphology, gram stains, catalase reaction, and CAMP tests. Antimicrobial susceptibility testing was performed using a modified Kirby–Bauer disc diffusion method. All collected data were entered in Epi info 4.6.0.2, then transferred and tabulated using SPSS version 20. Logistic regression analysis was used to see the association between variables. Finally, a *p*-value <0.05 was considered statistically significant.

**Results:**

In the present study, 279 pregnant mothers, aged between 15 to 38 years with a mean of 26.5 ± 4.5 years, were included. Of all participants, the highest proportion (120) (43.01%) were housewives. The overall carriage rate of *GBS* was 67 (24.0%). *GBS* colonization showed a statistically significant association with college and above levels of maternal education [AOR = 6.610, 95% CI (1.724–25.349), *p* = 0.01]. High susceptibility of *GBS* isolate was seen with Penicillin G & Chloramphenicol (92.5%), Ampicillin, Ceftriaxone (89.6%), Vancomycin (74.62%), and Erythromycin (77%). Relatively, *GBS* showed high resistance to Tetracycline (88%).

**Conclusion and recommendation:**

In this study, the overall prevalence of *GBS* colonization was 24.0%. College and above educational level was statistically significant with *GBS* colonization. This study aimed to draw attention to the management of *Group B Streptococci* in pregnant women by making *GBS* culture one of the routine diagnoses during ANC follow-up and to prevent infection with early detection.

## 1. Introduction

*Streptococcus agalactiae (GBS)* is a typical microbiota of healthy adults’ female genital tracts and anal areas, with the gastrointestinal tract acting as a natural reservoir and source of vaginal colonization. Pregnancy-related diseases such as urinary tract infection, bacteremia, chorioamnionitis, postpartum endometritis, preterm labor, preterm rupture of membranes, and perinatal transfer of the organism are all possible outcomes of maternal *GBS colonization. GBS’s* ability to rise from the lower genital tract and colonize the upper genital tract has been linked to intrauterine infection (Lämmler et al., 1995; Mengist et al., 2016, 2017; Liesse Iyamba et al., 2021).

*Group B Streptococcus* colonization of the genitourinary or gastrointestinal tract of pregnant women and its transmission to the infant during the labor and delivery process is the principal risk factor for early onset invasive *GBS* disease (Dadi et al., 2022). During pregnancy, approximately 10–30% of pregnant women are colonized with *GBS* in the vaginal and rectum area asymptomatically and 60% of their babies are infected through the birth canal (Melin and Efstratiou, 2013; Dadi et al., 2022).

Antibiotic resistance among *GBS* isolates has also been a concern due to the extensive use of intrapartum antibiotic prophylaxis to avoid early-onset *GBS* illness (Mengist et al., 2016, 2017; Leykun et al., 2021). *Streptococcus agalactiae* remain fully susceptible to penicillin as well as to most *β*-lactams, and penicillin remains the first-choice antibiotic to prevent *GBS*-EOD and to treat *GBS* disease. However, over the last two decades, resistance to macrolides and clindamycin among invasive isolates of *GBS* has increased from <5% to common resistance of 20–30% (Melin and Efstratiou, 2013; Dadi et al., 2022).

*Group B Streptococcus* colonization of pregnant mother is different in different countries. In the United States and Europe, *GBS* is the major cause of mortality and morbidity. It can be found in the vaginal microbiota of up to 30% of pregnant women and can be transmitted to the infant via a perinatal transmission (Centers for Disease Control and Prevention, 2007). A review that has specifically looked at the prevalence of maternal colonization with *GBS* indicated the estimated mean prevalence of *GBS* colonization was 17.9% overall and was the highest in Africa (22.4%) followed by the Americas (19.7%) and Europe (19.0%). However, studies from Southeast Asia had the lowest estimated mean prevalence (11.1%) (Kwatra et al., 2016; Mengist et al., 2016; Nishihara et al., 2017). Reduced by almost 80% in the United States, cases fell from 1.8 cases per 1,000 live births in the early 1990s to 0.23 cases per 1,000 live births in 2015 (Mengist et al., 2016). Evidence on maternal colonization prevalence remains sparse in African settings (Gizachew et al., 2019). In sub-Saharan Africa, the prevalence of *GBS* in Kampala, central Uganda was 3.9% (Tumuhamye et al., 2021), in the Democratic Republic of Congo, 23.07% (Liesse Iyamba et al., 2021), and in Kenya 20.5% (Jisuvei et al., 2020). In Sri Lanka, *GBS* vaginal colonization was 18% (Dilrukshi et al., 2021). Specifically in Ethiopia, studies have revealed maternal colonization ranges from 7.2% (Woldu et al., 2014) to 25.5% (Gizachew et al., 2019). Different studies conducted in Ethiopia indicated that Addis Ababa’s prevalence of *GBS* colonization among pregnant women was 14.6% (Assefa et al., 2018), in Jimma the prevalence of *GBS* colonization among pregnant women was 19.0% (Mengist et al., 2016), in Gondar the prevalence of *GBS* colonization among pregnant women was 25.5% (Gizachew et al., 2019), and in Nekemte the prevalence of *GBS* colonization among pregnant women was 12% (Mengist et al., 2017). Significant differences in the frequency of maternal colonization have been reported according to region, ethnicity, and socioeconomic characteristics (Kfouri et al., 2021).

Worldwide mortality from *GBS* colonization decreased from 12.7 million in 1990 to 6.3 million in 2013, but continuous effective measures should be made to decrease the mortality of newborns in developing countries (Nishihara et al., 2017). In Africa, the mortality rate is 4 times higher compared to America and Europe. So strategies for the prevention of *GBS* have a crucial role in mortality (Centers for Disease Control and Prevention, 2002; Nishihara et al., 2017).

*GBS* has the potential to thrive in a variety of diverse host environments (Plainvert et al., 2021). The problem is particularly immense in developing countries like Ethiopia that do not have quality microbiological laboratory facilities to isolate pathogens and determine their antimicrobial susceptibility pattern, in addition to the presence of fake drugs in circulation, and misuse of antimicrobials by health care providers, unskilled practitioners, and patients. Effective use of intrapartum antibiotic prophylaxis (IAP) reduces around 80% of early onset *GBS* disease. So a strategy on IAP evaluation for the prevention of EOD should be done in developed countries to decrease the burden of *GBS* disease, to develop a vaccine, or to prepare another preventive plan (Nishihara et al., 2017). There are many studies conducted in different cities of Ethiopia that show a high prevalence of the disease in mothers. However, there is no strategic plan developed to minimize the disease.

*GBS* infection is a challenging problem; much research has been done to show the prevalence of *GBS*, and its antimicrobial pattern has changed from place to place and from time to time. So the epidemiological data needs to be updated for a given place and time (Arain et al., 2015). Therefore, the main aim of this study will be to determine the prevalence of *GBS* bacteria in pregnant women and to carry out an antimicrobial susceptibility test in Wolaita Sodo Town, Southern Ethiopia. The result of this study may show the currently updated burden of the disease in Wolaita Sodo Town, Southern Ethiopia. This study could provide updated information for responsible bodies to formulate policies to implement prevention plans through universal screening for *GBS* in ANC units and effective use of prophylaxis to prevent early *GBS* infection.

## 2. Materials and methods

### 2.1. Study area

The study was conducted in Wolaita Sodo Town, which is located 327 km from Addis Ababa and 129 km from Hawassa. There are two general public hospitals, one governmental specialized hospital (WSUCSH), and three government health centers. In this study, two health facilities were used: WSUCSH and Wolaita Sodo Health Center (WSUCSH Co, 2022).

Wolaita Sodo Health Center under SNNPR Health Bureau provides outpatient services (ANC), follow-ups for adult OPD, pediatric OPD, a delivery service, TB patient follow-up, HIV counseling and screening, and health package services.

WSUCSH is a teaching and referral hospital of Wolaita Sodo University Health College, which started community service in 2009. It has about 500 beds, more than 300 health workers, and serves an average of about 1,000,000 patients annually (WSUCSH Co, 2022).

### 2.2. Study design and period

A health-facility-based cross-sectional study design was conducted at the ANC clinic of Wolaita Sodo University Comprehensive Specialized Hospital and Wolaita Sodo Health Center from June to August 2022.

### 2.3. Study population

All pregnant mothers who attended ANC follow-up at WSUCSH and Wolaita Sodo Health Center in Sodo Town and who were in their 35 to 37 weeks of gestational period were part of the study population.

### 2.4. Sample size determination

Sample size was determined using single population proportion formula considering the following assumptions: 95% confidence interval, 5% margin of error, and an assumed prevalence of 20.9% from the prevalence of *GBS colonization* among pregnant women in a previous study done Hawassa by Mohammed et al. (2010).

The following standard formula was used to calculate it.


$$n=\frac{Z\alpha^{2}P\left(1-P\right)}{d^{2}}$$

By using a 95% confidence level, the *Z* value was 1.96, with a 5% margin of error (*d*).

*P* = estimated prevalence rate = (20.9%); *α* = 0.05 (level of significance).

*n* = the required sample size.


$$n=\frac{\left(1.96\right)2\left(0.209\left[1-0.209\right]\right)}{{\left(0.05\right)}^{2}}=\frac{3.8416*\left(0.209*0.791\right)}{0.0025}$$

$$=254+\left(10\%\text{Non}-\text{response}\;\text{rate}\right):254+25=\underline{\underline{279}}$$

### 2.5. Sampling method

The study participants were enrolled using a systematic sampling technique until a sample size of 279 was achieved. The first participant was selected by the lottery method and by using the formula *K* = *N*/*n* (*K* = 424/279 = 1.52 ~ = 2 from Wolaita Sodo Health Center and *K* = 652/279 = 2.3 ~ = 2 WSUCSH) over a three-month period. Therefore, individual participants were selected randomly at every *K*th interval from two data collection institutions during the study period.

Then, the final study population of 279 was allocated proportionally to the size for each health facility based on their pregnant women visit as shown below. Pregnant women ANC visit in the year of 2021 from both Health facility data were shown below.

- *n*_WSUCSH_ = number of sample participants required from Wolaita Sodo University Comprehensive Specialized Hospital.
- *N*_WSUCSH_ = number of pregnant women who came to WSUCH from June 1, 2021 to August 30, 2021 GC, which was 652.
- *n*_WSHC_ = number of sample participants required from Wolaita Sodo Health Center.
- *N*_WSHC_ = number of pregnant women who came to Wolaita Sodo Health Center from June 1, 2021 to August 30, 2021, which was 424.
  - *n* = is the total sample size of the study, which was 279.
- *N*_Total =_ the sum of pregnant women both facilities from June 1 2021 to August 30, 2021, which was 1,076.

Therefore,

- *n*_WSCSH_ (from Wolaita Sodo Comprehensive Specialized Hospital) = (*n*/ *N*_Total_) − *N*_WSUCSH_.
  - *n*_WSUCSH_ = (279/1076) × 652 = 169, sample size was allocated to WSUCH.

Whereas

- *n*_WSHC_ (from Wolaita Sodo Health Center) = (*n*/ *N*_Total_) × *N*_WSHC_.
- *n*_WSHC_ = (279/1076) × 424 = 110 sample size was allocated to _WSHC_.

### 2.6. Data collection

The data on socio-demographic variables and other relevant information were collected using a predesigned and pretested structured questionnaire and by reviewing medical records. The questionnaire was adapted from other similar studies and initially prepared in English, translated to Amharic, and then translated back to English by another translator to check for consistency. Informed consent was obtained from each study participant after explaining the purpose and procedure of the study. The questionnaire was administered by the attending midwives and nurses to pregnant women with a gestational age between 35 and 37 weeks.

#### 2.6.1. Specimen collection

Specimens were collected as per the ACOG committee opinion and American Society for Microbiology (ASM) protocols. A vaginal-rectal swab was sampled from the mother at the point of ANC and labor by trained midwives using a sterile cotton swab. Using an aseptic technique by applying sterile cotton-tipped swabs in separate sterile tubes at the site of the rectum and vagina, the vagina swab from the mucosal secretions of the lower-third part was obtained. Thereafter, the rectum swab was carefully inserted into the anal sphincter and gently rotated to touch the anal crypts. Within 30 min the vaginal swab was placed in Amies transport media and within an hour of collection was transported to the Microbiology Laboratory of WSUCSH. Samples were transported in an ice box. All samples were cultured within an hour of arrival at the laboratory following standard bacteriological techniques (Leykun et al., 2021).

### 2.7. Laboratory procedures

#### 2.7.1. Culture and identification of *Group B Streptococci*

**Figure 1:**
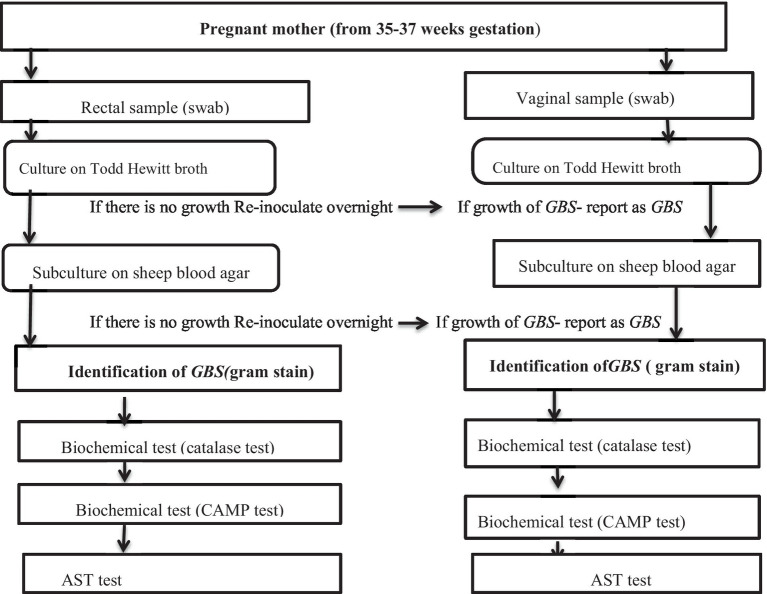
Flow chart diagram showing culture isolation, laboratory identification, and AST of *GBS*.

The swabs were inoculated in 1 mL Todd-Hewitt broth, an enrichment medium for *GBS*, supplemented with 10 μg/mL colistin and 15 μg/mL nalidixic acid to prevent contaminant growth. The samples were then incubated at 37°C aerobically for 18–24 h, sub-cultured onto sheep blood agar plates, and re-incubated at 37°C. After 24 h, the cultures were inspected for growth and all negative culture plates re-incubated for an additional 18–24 h and then re-observed. Plates that showed growth were identified by their characteristic appearance and biochemical tests such as catalase and CAMP testing; those with no growth were discarded or reported as negative (Figure 1).

CAMP testing was performed on sheep blood agar plate (SBAP) by streaking of *S. aureus* down the middle of SBAP and the test organism was then streaked perpendicularly to the *Staphylococcal* streak. The streaks did not touch. CAMP factor produced by *S. agalactiae* and β lysine produced by *S. aureus* act synergistically on SBAP to produce enhanced hemolysis. After incubation overnight under candle jar atmospheres, the SBAP was examined for an arrowhead-shaped zone of enhanced lysis Christie, Atkins, and Munch-Petersen (CAMP) factors. Those that were Gram-positive cocci in gram stain, catalase-negative in Biochemical tests, and CAMP positive were identified as *S. agalactiae* (Shiferawu et al., 2019).

#### 2.7.2. Antimicrobial susceptibility testing

Kirby Bauer’s disc diffusion technique was used to test the Antibiotic susceptibility (AST). The media used was Muller Hinton agar (MHA) supplemented with 5% sheep blood. From a fresh non-selective agar plate, pure colonies were selected and transferred to 5 mL Sterile normal saline and thoroughly mixed to make the suspension homogeneous. Turbidity was adjusted using a McFarland densitometer to match with a 0.5 McFarland Standard, then inoculated following the standard over the entire surface of an MHA plate using a sterile swab. Then, using sterile forceps, the antibiotic disks were placed on MHA 15 mm from the border and by considering the 24 mm distance between each disc and Zone of Inhibition was measured by the metric scale and reported as susceptible (S), intermediate (I), or resistance (R). Using the updated guidelines (CLSI, 2021), the following antibiotics disks were used for *Group B Streptococcus susceptibility*: Penicillin G 10 IU, Ampicillin 10, Erythromycin 15 Clindamycin 2, Ceftriaxone 30, Ciprofloxacin 5, chloramphenicol 30, Clindamycin 2, Vancomycin 30, and Tetracycline 30.

### 2.8. Data quality control

To assure the quality of the data, a pre-test was done and 5% of the total sample was out of the study area. Training was given to the data collectors on interviewing and recto-vaginal swab sample collection. Laboratory attendants were trained on how to clean, sterilize, and reuse laboratory materials. Investigators were trained on how to collect recto-vaginal swab samples by trained data collectors. The specimens were transported to the WSUCSH Central Laboratory within 30 min of collection in a cold chain (ice-box at 4°C) and immediately processed. Inoculums density for bacterial suspension for the antimicrobial susceptibility testing was standardized to 0.5. McFarlane Supervision was undertaken during the whole phase of the study period by the investigator and Medical Microbiologist. All culture media was prepared following the manufacturer’s instructions. All media was checked for sterility and performance. Reference strain *S. aureus* (ATCC-25923) was used as quality control throughout the study for culture and antimicrobial susceptibility testing. *E. fecalis* (ATCC-25212) and *S. pyogenes* (ATCC 19615) were used as a negative control for CAMP testing. To check the quality of the culture media and antimicrobial disks, control organisms were obtained from the EPHI (Ethiopian Public Health Institute). Samples were collected and processed aseptically using a standard operating procedure.

### 2.9. Methods of data analysis

Data were entered, cleaned, and processed into Epi 4.6.0.2 using SPSS version 20. Logistic regression analysis was used to see the association between variables. The “*p*” value was less than 0.05, which was considered statistically significant.

## 3. Results

### 3.1. Socio-demographic characteristics

A total of 279 pregnant women (from 35 to 37 weeks of gestation) were enrolled from June to August 2022, with a total response rate of 100%. The age of the study participants was between 15 to 38 years, with a mean age with SD of 26.5 (± 4.5) years. Most of the study participants (135) were aged between 25 and 29 years (48.4%). The majority of the study participants were married (249) (89.2%) and were urban residents (260) (93.2%). Of all participants, the highest proportion (120) (43.01%) were housewives, followed by civil servants (64) (22.9%), students (Garland et al., 2000) (17.9%), and merchants (Fatemi et al., 2009) (16.0%). A socio-demographic characteristic of study participants is given in Table 1.

**Table 1:** Socio-demographic characteristics among pregnant women at selected health facility of Wolaita Sodo Town, Southern Ethiopia from June to August 2022 (*n* = 279).

Socio-demographic characteristics	Categories	Frequency	Percentage (%)
Health institutions			
WSU Comprehensive Specialized Hospital		169	60.6%
Sodo Health Center		110	39.4%
Age groups	15–19	22	7.9%
	20–24	53	19.0%
	25–29	135	48.4%
	30–34	48	17.2%
	≥ 35	21	7.5%
Residence	Urban	260	93.2%
	Rural	19	6.8%
Educational status	Primary	166	59.5%
	Secondary	55	19.7%
	College& above	58	20.8%
Marital status	Married	249	89.2%
	Divorced	6	2.2
	Single	21	7.5%
	Widowed	3	1.1
Monthly income	1,300–3,000	221 ≥ 10,000	79.2
	3,100–5,000	35	12.5
	5,100–1,000	20	20
	≥ 10,000	3	1.1

### 3.2. Obstetric and clinical characteristics

Regarding obstetric and clinical characteristics of study participants, 173 (62.0%) were primigravida and the remaining 106 (38%) were multigravida. Of the study participants, 51 (18.3%) had a history of abortion and 13 (4.7%) women had a history of preterm labor. Among the 279 pregnant women included in the study, 58 (20.8%) were at a gestational age of 35, 146 (52.3%) were at a gestational age of 36, and the rest (75) (26.9%) were at 37 weeks of gestational age. History of hormonal contraceptive usage was reported by 223 (79.9%) of the study participants (Table 2).

**Table 2:** Obstetric and clinical characteristics among pregnant women at a selected facility of Wolaita Sodo Town, Southern Ethiopia from June to August 2022 (*n* = 279).

Variables	Categories	Frequency	Percent (%)
Number of gravidities	Primigravida	173	62.0
	Multigravida	106	38
Gestational age (in weeks)	35	58	20.8
	36	146	52.3
	37	75	26.9
History of contraceptive usage	Yes	223	79.9
	No	56	20.1
History of abortion	Yes	51	18.3
	No	228	81.7
History of preterm labor	Yes	13	4.7
	No	266	95.3
History of Preterm prolonged rupture of membranes	Yes	–	–
	No	279	100
Diagnosis of UTI during pregnancy	Yes	33	11.8
	No	246	88.2
Diagnosis of STI during pregnancy	Yes	3	1.1
	No	276	98.9
History of ANC visit	Yes	279	100
	No	0	0
History of any antibiotic use	Yes	0	0
	No	279	100
History of any chronic medical illness	Yes	16	5.7
	No	263	94.3

PROM, prolonged rupture of membrane; ANC, antenatal care; CMI, chronic medical illness; UTI, urinary tract infection; STI, sexually transmitted infections.

### 3.3. *Group B Streptococcus* colonization

The overall prevalence of *GBS* colonization among pregnant women at 35–37 weeks of gestation was 24% (67/279). The prevalence of *GBS* in the two health institutions was 37/67 (55.2%) from WSUCSH and 30/67 (44.8%) from Sodo Health Center.

### 3.4. Factors associated with maternal *Group B Streptococci* colonization

The assessment of the association of the socio-demographic, obstetric, and clinical characteristics with *GBS* colonization’s is demonstrated in Table 3. During the study period, a total of 279 mothers were screened for *GBS* colonization; *GBS* was confirmed in 67 (24.0%) of the study participants. In this study, the highest prevalence of *GBS* was observed in those aged between 30 and 34 years 12/48 (25%), housewifes 32/120 (26.67%), and participants with a college or above education status 15/58 (25.9%). Based on history of contraceptive usage, higher rates of *GBS* were observed in those with a contraceptive usage history 53/223 (23.8%) compared with non-users. Out of 67 *GBS*-colonized pregnant women, 34 (19.7%) were Primigravida and 33 (31.1%) were multigravida. Of the mothers who were at 37 weeks of gestation, 25 (33.3%) were culture positive for *GBS*. Women with no history of abortion had a 24.1% rate of colonization and women with a recent history of abortion had a rate of 23.5%. Variable candidates for multivariate logistics regression were selected by considering *p* < 0.25 from the bivariate model. Multivariable logistic regression analysis showed that mothers whose educational status was at the college level and above had a significant association with an increased risk of *GBS* colonization (*p* = 0.01). In this study, educational status, gravidity, maternal age, and gestational age showed an association with *GBS* colonization in binary logistic regression but not in multi-logistic regression.

**Table 3:** Bivariate and multivariate analysis of socio-demographic and obstetric factors among pregnant women at selected health facilities of Wolaita Sodo Town, Southern Ethiopian from June to August 2022 (*n* = 279).

Characteristics		*GBS*		COR 95% CI	AOR 95% CI	*p*-value
Variables	Categories	Positive (%)	Negative (%)			
Age group	15–19	6 (27.3)	16 (72.7)	0.403 (0.089, 1.835)	0.380 (0.078, 1.861)	0.240
	20–24	13 (24.5)	40 (75.5)	0.543 (0.137, 2.153)		0.385
	25–29	32 (23.7)	103 (76.3)	0.9589 (0.258, 3.560)		0.949
	30–34	12 (25)	36 (67.6)	0.192 (0.050, 0.741)	0.300 (0.072, 1.254)	0.099
	>35	4 (19.0)	17 (81.0)	1	1	
Educational status	Primary	49 (29.5)	117 (70.5)	1	1	
	Secondary	3 (5.5)	52 (94.5)	0.812 (0.413, 1.596)		0.545
	College & above	15 (25.9)	43 (74.1)	6.047 (1.642, 22.370)	6.610 (1.724, 25.349)	0.01
Occupational status	Housewife	32 (26.7)	88 (73.3)	0.770 (0.376, 1.578)		0.475
	Civil servant	14 (21.9)	50 (78.1)	1.276 (0.501, 3.245)		0.609
	Student	10 (20.0)	40 (80.0)	0.770 (0.317, 1.870)		0.564
	Merchant	11 (24.4)	34 (75.6)	1	1	
Gravidity	Primigravida	34 (19.7)	139 (80.3)	1	1	
	Multigravida	33 (31.1)	73 (68.9)	0.499 (0.286, 0.871)	1.761 (0.941, 3.296)	0.077
Gestational age	35	14 (24.1)	44 (75.9)	1	1	
	36	28 (19.2)	118 (80.8)	1.433 (0.671, 3.061)		0.352
	37	25 (33.3)	50 (66.7)	2.204 (1.166, 4.164)	1.509 (0.700, 3.254)	0.15
Contraception	Yes	53 (23.8)	170 (76.2)	0.946 (0.474, 1.890)		0.875
	No	14 (25.0)	42 (75.0)	1	1	
Abortion	Yes	12 (23.5)	39 (76.5)	1.184 (0.569, 2.462)		0.651
	No	55 (24.1)	173 (75.9)	1		
Pretermlabor	Yes	5 (38.5)	8 (61.5)	0.698 (0.208, 2.345)		0.561
	No	62 (23.3)	204 (76.7)	1	1	
UTI	Yes	6 (18.7)	26 (81.3)			
	No	61 (24.7)	186 (75.3)	0.835 (0.345, 2.020)		0.689

UTI, unitary tract infection; COD, crude odds ratio; AOR, adjusted odds ratio.

### 3.5. Antimicrobial susceptibility pattern of *GBS* isolates

The susceptibility patterns of *GBS* (*n* = 67) isolated from pregnant women were tested against nine antimicrobial agents (presented in Table 4). A high susceptibility rate of *GBS* isolate was seen for Penicillin G & Chloramphenicol (92.5%), Ampicillin and Ceftriaxone (89.6%), Vancomycin (74.62%), and Erythromycin (77%). Relatively, *GBS* showed high resistance to Tetracycline (88%), Ciprofloxacin (55.22%), and Clindamycin (23.9%). Regarding the antibiogram of *GBS* isolates, susceptibility to all antibiotics was observed in two (2.98%) *GBS* isolate and resistance to one or more antibiotics was observed in 65 (97.01%) tested *GBS* isolates. According to the study, the most active drugs for *GBS* isolates were Penicillin, Chloramphenicol, Ampicillin, and Ceftriaxone, with susceptibility results of 92.5, 92.5, 89.6, and 74.62%, respectively. Moreover, five (7.46%) isolates of *GBS* showed intermediate susceptibility to Erythromycins and Chloramphenicol, six to Vancomycin (8.9%), and two (2.98%) to Tetracycline.

**Table 4:** Antimicrobial susceptibility patterns of *GBS* isolated from pregnant women at selected health facility of Wolaita Sodo Town, Southern Ethiopia (*n* = 67).

Antibiotics with disc potency	Susceptible (%)	Intermediate (%)	Resistant (%)
Penicillin G (10 IU)	62 (92.5%)	–	5 (7.46%)
Ampicillin (10 μg)	60 (89.6%)	–	7 (10.4%)
Erythromycin (15 μg)	52/ (77%)	5/67 (7.46%)	10/ (14.92%)
Erythromycin (15 μg)	51/ (76.11%)	–	16/ (23.88%)
Vancomycin (30 μg)	50/ (74.62%)	6 (8.9%)	11 (16.41%)
Ceftriazone (30 μg)	60/ (89.6%)	–	7/ (10.4%)
Ciprofloxacin (5 μg)	30/ (44.77%)	–	37 (55.22%)
Chloramphenicol (30 μg)	62/ (92.5%)	5/ (7.5%)	–
Tetracycline (2 μg)	6/ (8.9%)	2/ (2.98%)	59/ (88%)

## 4. Discussion

The overall prevalence of *Group B Streptococcus (GBS)* in the present study among pregnant women was 24.0%. Such a result is comparable with studies worldwide, ranging from 10 to 30% in the USA, Kwatra et al. (2016) 6.5–36% in Europe (Nishihara et al., 2017), 7.1–16% in Asia (Centers for Disease Control and Prevention, 2007), and 11.9–31.6% in Africa (Arain et al., 2015). This study is also relatively similar to studies conducted in different parts of Ethiopia: 20.9% in Hawassa Health Centers (Mohammed et al., 2012), 19% in Jimma Hospital (Mengist et al., 2016), and 14.6% in different health centers in Addis Ababa (Assefa et al., 2018). The rate of *GBS* colonization in this study is lower than the studies conducted in Brazil at 28.4% (Melo et al., 2018) and South Africa at 30.9% (Bolukaoto et al., 2015).

The rate of *GBS* found in this study and some countries of Europe is comparable, for example in Italy two studies reported *GBS* rates as 17.9% (Melo et al., 2018) and 18% (Poyart et al., 2003). In Switzerland and Poland, positivity rates were 21% (Cockerill et al., 2012) and 17.2% (Blumberg et al., 1996) respectively. A study done in the Netherlands showed 21% (Flaherty et al., 2019). However, a lower *GBS* colonization rate was recorded from Istanbul and Elazin in Turkey giving 8% (Khan et al., 2015) and 8.7% (Assefa et al., 2018), respectively, while a study in Northern Greece reported the lowest rate of 6.6% (Valkenburg-Van Den Berg et al., 2006).

*GBS* colonization is an important cause of infection in pregnant women and is associated with adverse outcomes in their newborns; however, there have been limited studies available in Ethiopia (Assefa et al., 2018). It also has variable prevalence and susceptibility against commonly prescribed drugs in different geographic locations.

Providing adequate knowledge for pregnant women on *GBS* risk factors plays a crucial role in decreasing the morbidity and mortality related to maternal *GBS* infections. The geographical differences, variability in the sample size, and methods employed for *GBS* detection might possibly explain the disparities.

In this study, socio-demographic characteristics (age, residence, education status, marital status, income, and occupation) and Obstetrics and clinical characteristics (gravidity, gestational age, history of Preterm PROM, preterm labor, contraceptive use, history of abortion, UTI pregnancy, STI pregnancy, and any antibiotic) had no relation to the *GBS* colonization. Similar findings were reported from studies done in Italy that reported the *GBS* rate as 17.9% (Melo et al., 2018), Poland had a positivity rate of 17.2% (Blumberg et al., 1996). Having a college or above educational level was, however, significantly associated with maternal colonization (*p* = 0.01), with a similar finding shown in studies done in Poland (El Aila et al., 2010) and Bangladesh (Blumberg et al., 1996). Maternal age and gestational age were identified as risk factors for *GBS* colonization (Blumberg et al., 1996; Poyart et al., 2003; El Aila et al., 2010; Melo et al., 2018) in studies done before but no association was seen in the current study. In study done in Thailand reported that lower maternal age and lower gestational age were risks for colonization by *GBS* (Khan et al., 2015). The relationship between these factors and *GBS* colonization, however, showed marked inconsistencies.

In some studies, colonization increased with age (Cockerill et al., 2012), while other reports confirmed younger age groups showed the highest risk (Khan et al., 2015; Assefa et al., 2018). The possible reason for this difference seems to be seasonal differences globally, the availability of laboratory facilities for detecting *GBS*, and the length of study periods.

In this study, maternal age, gravidity, and gestational age showed association with *GBS* colonization in binary logistic regression, and college and above educational level showed an association on multi-logistic regression.

In the current study, the susceptibility pattern of *GBS* isolates to Penicillin (92.5%), Vancomycin (74.62%), ampicillin (89.6%), Ceftriaxone (89.6%), Chloramphenicol (92.5.%), Erythromycin (77%), and Clindamycin (76.11%) is comparable with previous studies conducted in different countries, in which similar records were found from Mekele, Ethiopia (Alemseged et al., 2015), the USA (Rausch et al., 2009), Canada (Ayata et al., 1994), and Lebanon (El-Kersh et al., 2002).

However, high resistance was observed in Tetracycline (88%), Ciprofloxacin (55.22%), and Clindamycin (23.88%). Similar records were found in Lebanon (El-Kersh et al., 2002), the USA (Rausch et al., 2009; Strus et al., 2009), Mekele, Ethiopia (Alemseged et al., 2015), and Canada (Ayata et al., 1994). Erythromycin (14.92%), Vancomycin (16.41%), Ceftriaxone and Ampicillin (10.4%), and, *GBS* resistant with reduced Penicillin susceptibility have been detected. Penicillin is the first agent for the prevention and treatment of GBS infections; however, nowadays GBS strains with reduced susceptibility to Penicillin have been reported periodically as seen in different study previously like also this study.

To prevent *GBS*, Erythromycin and Clindamycin are alternative antibiotics to Penicillin, especially for pregnant women with allergies who are at a high risk of anaphylaxis.

The increase of *GBS* strains resistant to Erythromycin and Clindamycin is complicating the management of pregnant women who are allergic to Penicillin (Valkenburg-Van Den Berg et al., 2006). In contrast to reports from many other countries, the highest susceptibility in the present study was seen to Erythromycin (77%) and Clindamycin (76.11%), and only a few isolates were resistant to these drugs. In this study, 15% Erythromycin and 24% Clindamycin resistance was reported, which is similar to studies done in Ethiopia, Gondar, showing a 22.7% resistance to Erythromycin and 17.6–18.2% resistance to Clindamycin (Schmidt et al., 1989). Also in South Africa, 17.2% Clindamycin and 21.1% Erythromycin resistance were reported (Verani et al., 2010). In Italy and the USA, Clindamycin resistance was reported at 17.6% (Savoia et al., 2008) and 21.0% (Fatemi et al., 2009), respectively, which is comparable to our study.

Generally, in contrast to this study, worldwide studies have reported a high resistance rate to Erythromycin, which ranges from 18 to 54% (Blumberg et al., 1996). Absence or low antibiotic resistance of *GBS* strains in the present study may indicate the suitability of Penicillin, Ampicillin, and Chloramphenicol for Ethiopia to prevent *GBS* until a vaccine is available on the market.

Similar to the present study, low levels of resistance to Erythromycin was reported in Australia (6.4%) (Assefa et al., 2018), Brazil (4.1%) (De Steenwinkel et al., 2008), on the Thai-Myanmar border (8.5%) (El Aila et al., 2010), and France (4%) (Blumberg et al., 1996). No resistance to Chloramphenicol was observed in this study and two (7.5%) and three (10.4%) of the isolates showed resistance to Penicillin and Ampicillin, respectively.

High resistance to Tetracycline (88%) in this study was reported and similar reports from other countries, such as Brazil (83%) (Valkenburg-Van Den Berg et al., 2006), Australia (85.9%) (Savoia et al., 2008), Kuwait (89.5%) (Rausch et al., 2009), Canada (89%) (Ayata et al., 1994), and Greece (85%) (Tsolia et al., 2003) also reported.

CDC-approved patients can take Penicillin or Ampicillin if they are not allergic to Penicillin. Clindamycin or Vancomycin is the drug of choice for those who have a major Penicillin allergy and Ceftriaxone for a minor allergy to Penicillin (YektaKooshali et al., 2018). It is difficult to develop a vaccine for *GBS* due to its multiple serotypes and variations in geographical locations (Garland et al., 2000). In addition, resistance to Clindamycin and Erythromycin, which are first-line drugs for those with a penicillin allergy, has increased rapidly (Garland et al., 2000).

Resistance to Erythromycin ranged from 7 to 40% and Clindamycin from 3 to 26.4%; this was related to some serotypes (Fatemi et al., 2009; Tsui et al., 2009; Khan et al., 2015). Inappropriate use of antimicrobial drugs leads to high drug resistance. In Ethiopia, people can easily go to pharmacies without a prescription to buy antibiotics and this type of antibiotic use might responsible for the high drug resistance rates observed currently.

### 4.1. The strengths of this study

In this study, a more valid method was used to identify *GBS* colonization. The use of THB (Todd-Hewitt broth), a primary selective broth media for isolation of *GBS* that consisted of 10 μg/mL colistin and 15 μg/mL nalidixic, as well as the use of antibiotics in the primary media to selectively isolate the bacteria, makes our isolation able to indicate maximum carriage rate.

### 4.2. Limitations of the study

- No serotyping was done and only disc diffusion was used for the antibiotic susceptibility test.
- There was a failure to assess the outcome on neonates whose mothers were detected to be colonized by *GBS* in the study.

## 5. Conclusion

The prevalence of *GBS* in the current study was 24.0%. Among 279 pregnant women, the carriage rate of *GBS* was highest among those aged 30–34 years. Having a college or above level of maternal education was significantly associated to maternal colonization in the current study [AOR = 6.610, 95% CI (1.724–25.349), *p* = 0.01]. The highest susceptibility was shown for penicillin and chloramphenicol (92.5%). High resistance was observed against Tetracycline (88%) and Ciprofloxacin (55.22%). The overall prevalence of *GBS* in the current study is high. Therefore, there is a need for screening of pregnant mothers near term delivery and to determine their antibiotic susceptibility so as to set appropriate intervention mechanisms. Early diagnosis, treatment, and proper management is very important to reduce *GBS* infection of neonates and newborns.

## Data availability statement

The original contributions presented in the study are included in the article, further inquiries can be directed to the corresponding author.

## Ethics statement

The studies involving humans were approved by the Institutional Review Board of College of Health Sciences and Medicine, Wolaita Sodo University. The studies were conducted in accordance with the local legislation and institutional requirements. The participants provided their written informed consent to participate in this study.

## Author contributions

AK: Conceptualization, Data curation, Formal analysis, Investigation, Methodology, Software, Supervision, Validation, Visualization, Writing – review & editing. BG: Conceptualization, Data curation, Formal analysis, Investigation, Methodology, Project administration, Resources, Software, Supervision, Validation, Visualization, Writing – original draft. SS: Conceptualization, Data curation, Methodology, Project administration, Supervision, Validation, Visualization, Writing – review & editing. WW: Conceptualization, Software, Supervision, Validation, Writing – review & editing. TS: Data curation, Formal analysis, Software, Supervision, Validation, Writing – review & editing.
